# Training for “Worst-Case” Scenarios in Sidestepping: Unifying Strength and Conditioning and Perception–Action Approaches

**DOI:** 10.1186/s40798-023-00566-8

**Published:** 2023-04-05

**Authors:** Daniel Kadlec, Matt Miller-Dicks, Sophia Nimphius

**Affiliations:** 1 School of Medical and Health Sciences, Centre for Human Performance, Edith Cowan University, 270 Joondalup Drive, Joondalup, WA 6027 Australia; 2 School of Sport, Health Exercise Science, University of Portsmouth, Portsmouth, UK

**Keywords:** Sidesteps, Movement strategy, ACL

## Abstract

Sidesteps can impose high demands on the knee joint and lead to non-contact anterior cruciate ligament (ACL) injuries. Understanding how different constraints shape an athlete’s movement strategy and the associated joint demands can help design training interventions to increase injury resilience. Motor capacities, such as muscular strength and power, act as boundaries for the safe execution of perceptual–motor skills and co-determine the emergence of unique movement strategies. Increasing single- and multi-joint strength enables a broader solution space for movement strategies and increases load tolerance. Manipulating task constraints during sidesteps can be used in the training process to systematically expose athletes to increasing demands (on the knee joint or any joint or structure) in preparation for “worst-case” scenarios. In particular, the type and timing of information available influence the preparation time, subsequently affecting the movement strategy and the associated magnitude of external knee joint loading (e.g., knee valgus moment). While an athlete’s perceptual–cognitive skills contribute to the preparation time during in situ scenarios, attempts to further improve those skills with the aim of increasing athlete preparation time prior to “worst-case” scenarios are yet to demonstrate conclusive evidence of transfer to on-field situations. Therefore, in the current article, we reflect on the impact of different interacting constraints that influence the execution of sidesteps during in situ scenarios and impose high demands on the knee joint. Subsequently, we discuss how an integrated perspective, drawing on knowledge and perspectives from strength and conditioning and perception–action, may enhance an athlete’s ability to withstand “worst-case” scenarios and adapt to perform varied movement executions when sidestepping.

**Supplementary Information:**

The online version contains supplementary material available at 10.1186/s40798-023-00566-8.

## Key points

1. Single- and multi-joint strength acts not just as a boundary but rather as a catalyst for distinct and unique movement solutions, as utilizing the “strongest” set of motor capacities reduces the relative energy systems cost and facilitates the emergence of dominant movement strategies that may remain consistent even with variations to the task.
2. Exposing athletes to exploratory learning conditions or variable practice may allow them to explore the range of their new motor capacities to translate improved muscular strength into calibrated movement strategies.
3. The efficacy of training perceptual–cognitive skills in isolation or in non-representative settings to improve in situ performance with the goal to allow for more preparation time prior to “worst-case” scenarios remains questionable.
4. The sources of information athletes are reacting to (generic information [e.g., flashing arrow or light] or specific information [e.g., video of opponent]) affect the demands on the knee joint and can be used during unplanned sidesteps to progressively overload knee joint tissues.

## Introduction

Motor capacities are integral to motor skill and performance from both strength and conditioning [1] and skill acquisition [2] perspectives (see Table 1 for definitions of key terms used in this article). It follows that improving motor capacities, often focusing on muscular strength, has a broad and well-supported evidence base for the enhancement of athletic performance and reduction in injury risk [1, 3]. Although increasing muscular strength is related to improvements in gross motor skills [4], the timeframe and relationship between these two facets of performance are not well understood, especially for more complex motor tasks [5]. The link between these two research domains has been alluded to in proposals, which emphasize the need to consider *“learning to use one’s newfound strength*” [1] as the critical bridge between motor capacity and motor skill [6, 7]. Therefore, using a training process that integrates motor capacity building in tandem with skill development to maximize the transfer of training is essential for enhancing athletic performance and mitigating injury risk.

**Table 1:** Definitions of key terms. Many terms in this manuscript are used across disciplines (e.g., strength and conditioning, motor learning and skill acquisition, biomechanics) and used in different contexts [8]

Motor skill	The ability to execute a pattern of behavioral elements in proper relation to certain environments [9]
Motor capacity	Adapted from a prior definition of motor capacity as “*what a person can do in a standardized, controlled environment*” [10], in the current context, this will be delimited to one’s ability to apply force in a standardized, controlled environment or a measure of strength
Perceptual–cognitive skill	The ability to identify and process key environmental information without the necessity to execute a motor skill for a particular task
Perceptual–motor skill	The ability to exploit key environmental information during the coordination of motor skills for a particular task
Joint loading	Joint power (W) reflects the rate of energy or work (J) generation (or absorption) over time and acts as a proxy for the term “joint loading” throughout this article

**Fig. 1:**
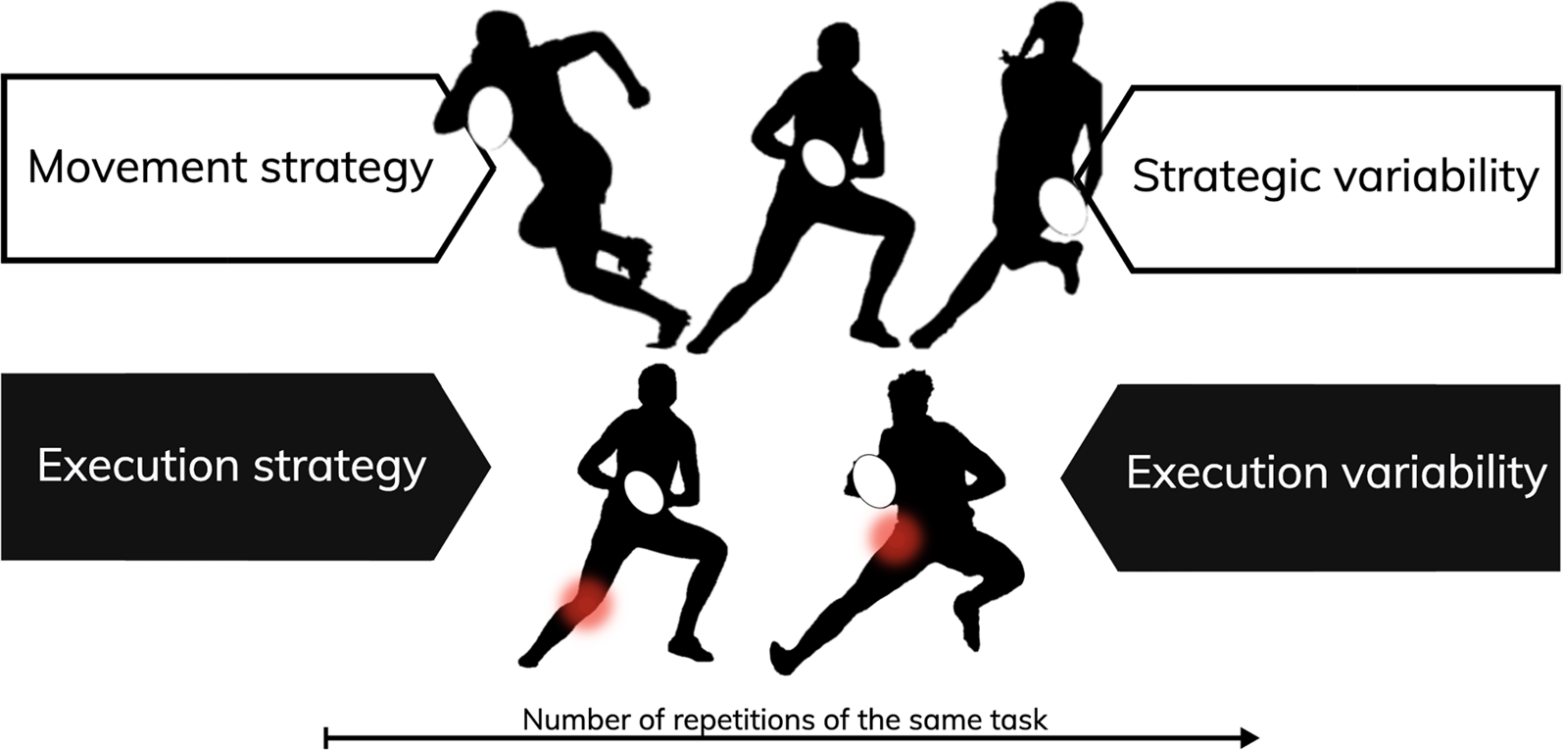
Overview of key definitions based on Cowin et al. [25]. **Movement strategy:** The kinematically or kinetically distinct and classifiable motor solution used during the execution of the task. For example, kinematically or visually defined movement strategies may be categorized as a crossover cut or sidestep. In contrast, kinetically defined movement strategies may be described categorically as “knee-dominant” or “hip-dominant” as indicated by the red circles. Further, some movement strategies may be defined by clustering a combination of kinematic and kinetic variables depending on the author's or practitioner's definitions. **Strategic variability:** *“Describes the different approaches or methods of movement used to complete a task.”* Strategic variability is discussed and described over multiple efforts or trials of the same task or how many different movement strategies are used to complete the same task. **Movement execution:** The magnitude and distribution of joint kinetics or kinematics of a performed trial or effort within a movement strategy. **Execution variability:** *“Describes the intentional and unintentional adjustments of the body between repetitions within the same strategy”*

Therefore, the following terms include definitions aligned with their use in the current manuscript (Fig. 1)

Understanding the relationship between motor capacities and motor skills (e.g., sprinting, jumping, changing direction, throwing, striking) has become a topic of debate in the strength and conditioning literature emphasizing perception–action processes [11]. For example, recent strength and conditioning literature has suggested that movement assessments should be representative of sports situations and reflect sport performance contexts [11]. However, representative design creates challenges for systematic control of the experimental setting and the reliability of testing procedures [12]. Further, if one aspires to design assessment settings entirely representative of sports performance, the situations sampled should arguably amount to the sports themselves [13, 14]. Due to this methodological complexity and difficulty in (defining and) quantifying a) agility performance and b) the transfer to in situ scenarios, research is lacking to provide practitioners with an actionable training framework. Early agility research used generic stimuli (lights on a target board) [15], before replacing the light response conditions with sports-specific video [16], face-to-face human “opponents” [17] as well as many other variations on the generic stimuli such as arrows [18] or colored lights [19]. More recently, researchers have studied multiple changes of direction in response to 2D variations of sports-specific movements [20] and 3D stereoscopic images with one and two defenders [21]. Despite the highlighted development of methods, it could be argued that all these assessments may still fail to provide representative testing conditions [22]. Sidestepping is beneficial to athletic performance but is a high-risk, high-reward movement due to its association with anterior cruciate ligament (ACL) injury risk [23] as evidenced by the number of non-contact ACL injuries experienced in team sports [24, 25]. It is, therefore, an area of particular interest within sports medicine research.

In this perspective article, we will consider sidestepping as a practical case for describing a theoretical “worst-case” scenario, considering task demands elicit high joint loads (e.g., knee valgus moment) threatening the system’s structural integrity. We define a “worst-case” scenario as the emergence of an extreme internal response threatening tissue integrity due to a complex combination of physical/organismic and contextual factors. We reflect on the implication of different interacting constraints when executing sidesteps within in situ scenarios imposing high demands, particularly on the knee joint. Since ACL injuries occur during instances of high acceleration or deceleration of movement within brief moments of time, the resultant strain on the ACL or, more generally, around the knee joint is dependent upon the movement velocity with respect to time [26, 27]. As such, joint power (W) reflects the rate of energy or work (J) generation (or absorption) over time and acts as a proxy for the term “joint loading” throughout this article. Further, we will discuss how utilizing such constraints can increase an athlete’s execution variability, or in other words, enhance the number of different executions used within a single movement strategy across repetitions of the same task and withstand “worst-case” scenarios (Fig. 1). Finally, we evaluate the importance of perceptual–cognitive skills to sidestep mechanics and review the effectiveness of various methods to improve perceptual–motor skills.

## Implications for Different Interacting Constraints on Knee Joint Loading

### Does Motor Capacity Influence the Movement Strategy?

Human movement is a function of the intrinsic and extrinsic constraints described in Newell’s theoretical model of constraints on developing coordination: organismic (individual), environmental, and task constraints [2]. A central proposal is that an athlete’s individual and unique movement solution emerges from the interacting constraints. Thus, even during pre-planned sidestepping, athletes execute movements differently with distinct joint loading profiles indicated by the magnitude and distribution of joint kinetics [28, 29, 30]. Franklyn-Miller et al. [30] demonstrated three participant joint clusters based on joint kinematics and kinetics when sidestepping. More broadly, cluster 1 did more joint work at the knee, cluster 2 at the hip, and cluster 3 did more work at the hip and ankle. Further, the participant cluster with greater joint energy absorption or joint work at the knee during the ground contact phase of the cutting step also had higher knee valgus moment and internal rotation moments. A lab-based surrogate of these loading patterns proposed that the knee-based cluster group had increased ACL strain [15, 31]. The “knee-dominant” groups, or groups with greater work done at the knee, presented significantly altered thorax and pelvis kinematics, especially in the frontal plane, during the cutting step [28, 29] and the penultimate and ante-penultimate steps compared to the “non-knee-dominant” groups [28]. These studies [28, 29, 30] were conducted with pre-planned or anticipated tasks, which highlights that even under no additional temporal or spatial constraints, athletes exhibit individual or categorical (movement strategy) patterns of joint loading. The emergent movement execution, or how they perform the motor task, directly determines the demands imposed on the joint(s). However, as motor capacities were not quantified, it remains unknown if or how the motor capacities of the athlete facilitate the emergence of specific execution clusters [28, 29, 30].

**Fig. 2:**
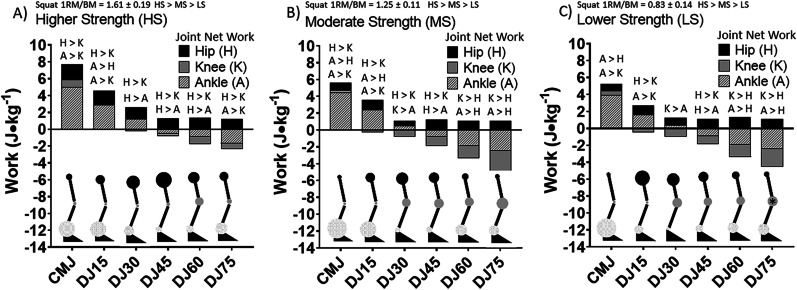
Comparison of net work (J) for the hip, knee and ankle between **A** higher strength (HS), **B** moderate strength (MS) and **C** lower strength (LS) groups for the countermovement jump (CMJ) and drop jumps (DJ) from 15, 30, 45, 60 and 75 cm. Significances indicated at *p* ≤ 0.05. H > K, A > K, A > H, H > A, K > A, K > H significances indicated at *p* ≤ 0.05. *Indicates the knee net work (J) was significantly great in LS in comparison with HS at *p* ≤ 0.05. The size of the circles at each joint is a scaled quantitative representation of the relative amount of net work (J) performed by that respective joint. Reproduced from McBride and Nimphius [39], with permission

An individual’s motor capacities, such as strength and power, influence the emergence of movement and, therefore, the associated joint loading pattern [32, 33, 34]. Manipulating any constraint consequently affects the athlete’s movement strategy. Single- and multi-joint strength changes after training interventions, such as resistance or plyometric [32]. Muscular strength often differentiates athletes of various playing levels and shares a moderate-to-strong relationship with motor skill and injury risk [35, 36]. Increased single- and multi-joint strength theoretically allows the athlete to express a greater solution space of movement strategies for a given task [2] and maintain a greater strategic variability when task demands increase [37]. Alternatively, increased single- and multi-joint strength may require higher eccentric demands before shifting to a strategy that results in greater net energy absorption at the knee [33]. In Fig. 2, it is noted that higher strength (> ~ 1.6 × bodyweight) was associated with higher eccentric demands (height of drop jump) before negative net work was done at the knee. Therefore, the evidence supports the proposition that developing motor capacities may facilitate changes in movement strategy. However, it is noted that having adequate strength or motor capacity does not guarantee coordination and control [38], and therefore, other factors interact with motor capacity to determine movement strategy.

### What are We Preparing Athletes for?

The information outlined above indicates that athletes may have a preferred movement strategy for sidestepping, which is in accordance with theories from ecological dynamics (for further reading, see Warren [40] and Yamamoto et al. [41]). The emergent movement solution is affected by the demand of the task and constrained by the motor capacity and control of the athlete. However, a single movement solution is not advantageous for sports or for a motor skill that varies relative to unpredictable game demands, such as unplanned sidestepping. Being adaptable as a means to solve a motor task, or to have adequate execution variability [8], increases the likelihood that athletes succeed “despite” the scenario or position they are in at a given moment. Lacking motor capacity (e.g., strength) in a particular segment may reduce the execution variability. Specifically, leg strength and trunk control may affect the strategy and execution during more demanding sidestepping (e.g., increasing angle or velocity of performance) [20, 42, 43].

Inconsistent results [44, 45, 46] concerning the influence of isolated parameters (e.g., knee valgus angles or moments at specific time points during athletic tasks) on injury risk highlight the need to identify underlying movement strategies to increase the understanding of the magnitude and distribution of joint loading and their association with injury risk [47]. For example, quantifying knee valgus moments in isolation in a particular task provides insufficient predictive information regarding injury risk [47]. Consequently, individual movement strategies for a given task can differ between athletes and impose distinct demands on the system. Understanding how modifiable constraints (e.g., single or multi-joint strength) influence the emergence of movement executions can help the practitioner design effective drills or individualized exercises to increase an athlete’s tolerance to “worst-case” scenarios. In our understanding, such “worst-case” scenarios accompanied by biomechanically compromised positions during in situ scenarios may eventually happen. Hence, preparing an athlete for such “worst-case” scenarios is more sport relevant than a “movement solution avoidance” approach commonly recommended in the literature (e.g., minimize the distance between plant foot and center of mass (COM), reduce lateral trunk flexion, or increase knee flexion at initial contact) [48].

### Do Athletes Use the Same Strategy Repeatedly?

If a movement strategy is repeated consistently, this will strain certain tissues dictated by the movement strategy, implying that a subset of tissues will not be strained [49]. Motor capacities act as boundaries that co-determine the safe execution of motor skills relative to changing environmental demands. The inherent between-subject difference in specific motor capacities [50] and the ability to utilize them within a particular task dictates the movement execution [51]. Thus, single- and multi-joint strength acts not just as a boundary but rather as a catalyst for distinct and unique movement solutions. Utilizing the “strongest” set of motor capacities may reduce the relative energy systems cost and facilitate the emergence of dominant movement strategies [7, 34, 39]. These may remain consistent even with variations to the task [52]. Complementary to this view is an affordance-based control framework, proposing that skilled performance necessitates that an individual’s motor capacities are scaled or calibrated relative to environmental features [53, 54, 55]. Hence, insufficient muscular strength may limit the possible number of movement strategies and enforce a repetitive straining pattern on the system. Reduced execution variability represents a less adaptable system, which can either contribute to an increased injury risk due to an accumulation of chronic local tissue strain exceeding tissue tolerance thresholds over time [56, 57] or the results of a previously suffered injury [58, 59, 60]. Repeated loadings of sufficient magnitude and frequency can disturb physiological repair mechanisms and cause pathological tissue degeneration. One single loading exceeding the failure threshold applied over a short timeframe can result in a traumatic injury [49, 61]. This is particularly the case when executing a repetitive “knee-dominant” strategy, which can increase injury risk, as evidenced by the number of non-contact ACL injuries experienced during sidestepping [24, 25].

Task demands also play a crucial role in the emergence of possible movement solutions. The utilized movement strategy combined with the task demands determine the resultant loading on the system as quantified by ground reaction forces (GRF) and the magnitude and distribution of intra-individual joint loading [62]. Alteration of the task demand or the movement strategy can elicit changes in GRF and joint-specific loading strategies in different motor tasks [63, 64, 65]. When sidestepping, factors such as the entry velocity, the change of direction angle, trunk alignment, and the time to anticipate the cutting direction dictate the task intensity and affect knee joint loading (e.g., knee valgus moment) [66]. Such changes in the demands imposed may be further affected by fatigue, postural variation, and previous injuries [62, 63, 64, 65]. However, adequately distributing the imposed demands across several joints can be compromised by increasing task intensities. Various measures of trial-to-trial execution variability, including kinematic, kinetic, and electrical muscle activity, decrease with a concomitant reduction in the number of available movement strategies with increasing task demands (e.g., drop height with single leg landings) [64, 65].

During unplanned sidesteps, the execution variability of kinematic variables increases, as all degrees of freedom must be coordinated and integrated to achieve the desired outcome under high demands, compared to pre-planned sidestepping [67]. However, as quantified via knee valgus moment, the knee joint loading only differs significantly in ~ 10% of the stance phase in the frontal plane between pre-planned and unplanned actions [67]. Therefore, despite significant increases in execution variability of kinematic variables, minimal differences in knee joint kinetics occurred. This creates a unique dichotomy that requires further research to elucidate whether the emergent movement execution variability in kinematics has minimal influence on changing the kinetically defined movement strategy. Or, in other words, is an athlete with a “knee-dominant” strategy more likely to solve many motor tasks with the same kinetically defined movement strategy? If what a practitioner sees (kinematics) has a lesser impact than what the athlete withstands (kinetics), this may explain research demonstrating that kinematic-focused screens for “ideal” movement have limited utility in predicting injury [68].

**Fig. 3:**
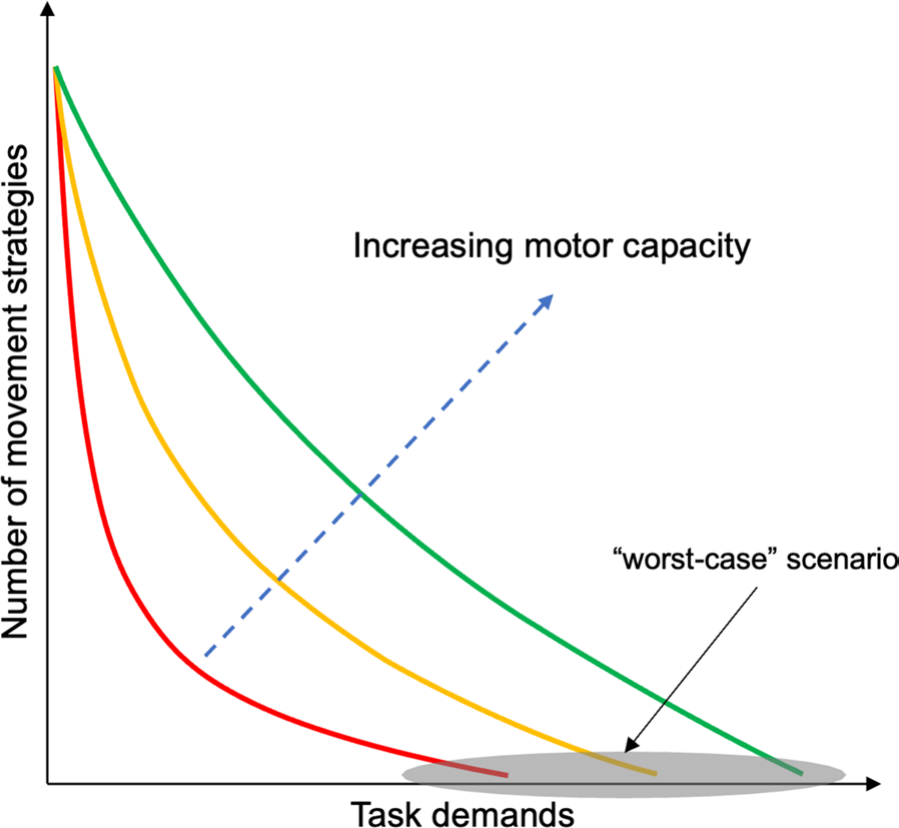
The theoretical relationship between task demands and the number of possible movement strategies with increasing motor capacities

The relative task demands dictate the solution space of possible movement solutions across sports situations, including sidestepping (Fig. 3). However, drawing implications from the mechanical task demand as described by GRF or segmental kinetics (i.e., joint moment or power) is insufficient to determine the demand for an individual athlete. The demand of a task for an individual is influenced by their motor capacities and control and subsequently determines the magnitude and distribution of joint loading [7, 32, 33, 34, 64, 65]. Hence, it is not the absolute load but the load relative to the athlete’s motor capacity and control that dictates how the system responds – either via adaptation or injury [69]. When executing the same task, athletes with differences in motor capacity or control can display unique execution strategies with distinct biomechanical or physiological responses [33, 70]. Athletes with less flexible movement solutions [71] exhibit higher GRFs during a bilateral jump-landing task in different conditions and a heightened acute physiological stress response than skillful movers [72]. Similarly, weaker athletes experience greater knee joint loading when performing drop jumps from increasing drop heights than stronger athletes (Fig. 2). Therefore, lacking sufficient motor capacities reduces the ability of the system to modulate the energy during the task and, therefore, may increase the demands on the knee when performing the athletic task [33, 70, 71]. While the movement outcome is a combination of the task, environment, and the individual, lacking muscular strength inevitably limits the solution space of possible movement strategies and reduces execution variability, thus potentially facilitating a repetitive straining of certain tissues.

**Fig. 4:**
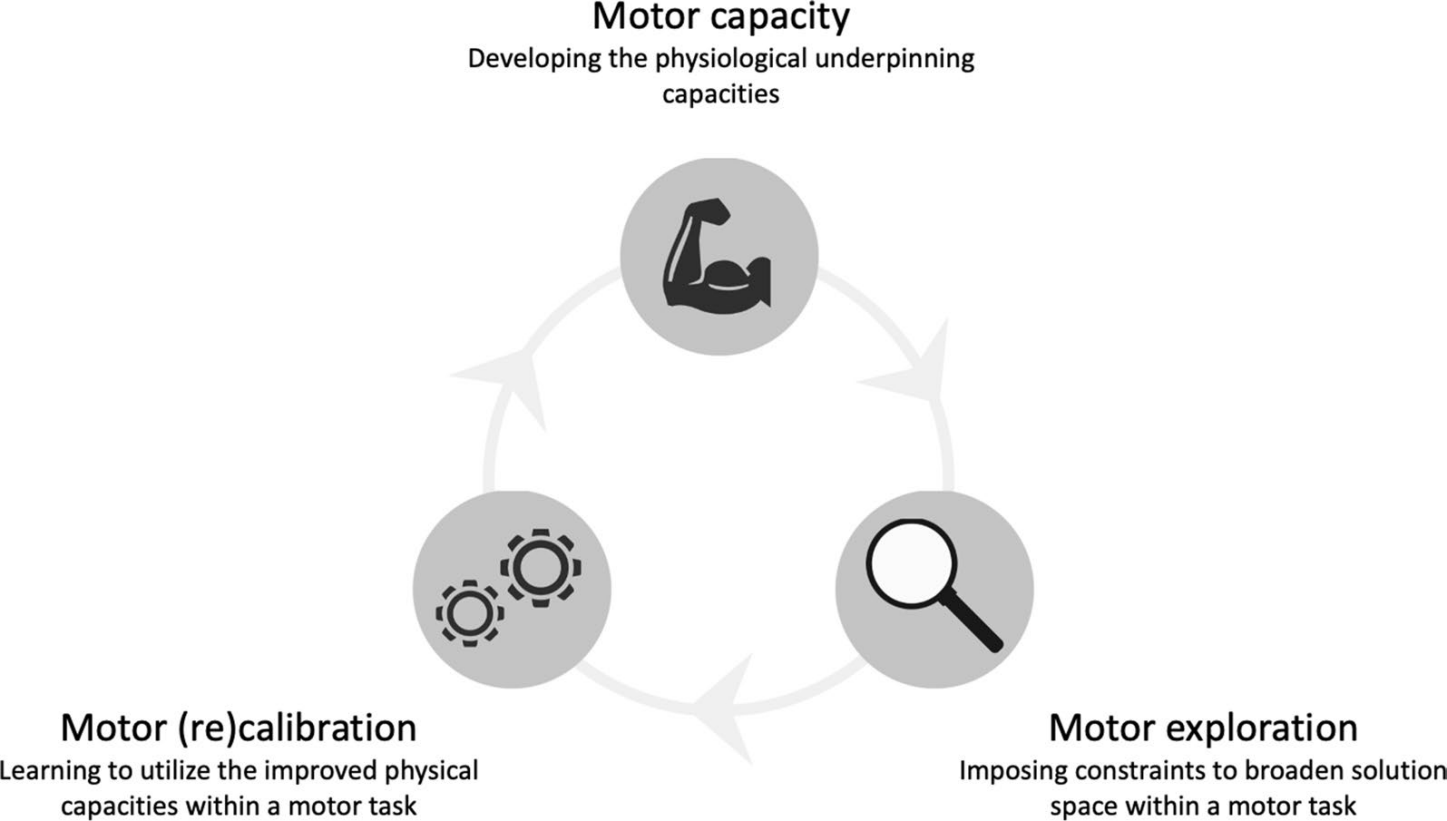
Continuous iterative (re)calibration framework of learning to transfer and express strength gains in movement

Practitioners should consider that athletes may have dominant movement strategies when planning training and designing drills. Unless task or environmental constraints alter the movement strategy, it is plausible that athletes utilize a dominant movement strategy independent of the task demands [70]. However, even with increased muscular strength, as seen after resistance or plyometric training interventions, athletes need to learn to utilize the now-improved motor capacities. This process may be mainly facilitated through exploratory learning conditions [73]. In other words, athletes may need to be exposed to variable practices that allow them to explore the range of their new motor capacities to translate improved muscular strength into calibrated movement strategies [74]. Without adequate practice, athletes may fail to broaden the solution space and, therefore, may not (re)calibrate to their newfound strength and fail to adequately adopt new movement strategies and executions within a motor task [1, 74, 75]. This perspective implies that sufficient motor capacities are not enough if coaches want to adequately prepare athletes for in situ demands (Fig. 4).

Athletes should be prepared for possible “worst-case” scenarios to minimize injury risk. Preparation is essential since an athlete can be forced into situations where the dominant or trained movement strategy, and related tissues accustomed to tolerating the demand of the strategy, may not be a viable or desired solution. In such “worst-case” scenarios, an underused movement strategy must be executed, placing demand on potentially under-trained joints and tissues. Further, athletic success is desirable despite the scenario and position the athlete is exposed to. The process outlined in Fig. 4 enables the physiological development of tissues or structures and the enhancement of coordination and control to execute various movement strategies.

## Constraints as a Tool to Increase Movement Solutions

### How Does the Anticipatory Information Available to an Athlete Affect Joint Loading?

Variation in the stimuli or available information provides different spatiotemporal conditions for coordinating movements during sidestepping. The information (i.e., lights, video, or another athlete) and the subsequent response of an athlete have frequently been termed perception–action coupling. The origin of this proposal is attributed to Gibson’s [76] ecological approach, which proposes that the perceptual systems are active, enabling the detection and creation of information, which is used in the control of movement in an ongoing, cyclical manner [77]. Considered practically, perception–action is exemplified by an athlete sidestepping to displace the positioning of an opposing defender to create the time and space needed to run past the defender. Thus, attempts to quantify an athlete’s (passive) response to a video or light stimulus are arguably not an accurate translation of Gibson’s [76] ecological approach, as the actions of performers relative to a light or a video do not enable the generation of new information in a cyclical perception–action manner. Therefore, the action in a “lab-based” response lacks any consequences for the athlete, inviting them to “gamble” their motor response due to the absence of an interaction or consequence. Hence, unless the athlete coordinates their actions relative to at least another athlete who actively engages in a representative (sport-specific) setting, the perception–action processes underpinning movement strategies may not represent in situ demands [78, 79]. However, anticipatory information and timing can be considered for a different reason than enhancing representativeness.

Sidestepping in a laboratory setting can be executed as pre-planned movements (the athlete knows in advance the desired cutting direction) or unplanned movements (the athlete has to act relative to a sudden change in information). The sources of information in unplanned drills can be considered either generic or specific. Generic information comprises flashing arrows or lights indicating the required direction of movement. In contrast, specific information includes either a video or a three-dimensional (3D) projection (i.e., virtual reality) of one or more “opponents” presented in a simulated sport context. Each of these experimental settings elicits distinct loading profiles of the knee joint (e.g., knee valgus moment) [21]. Sidestepping within unplanned conditions elicits different kinematics of the lower limbs and trunk during the ground contact phase than in a pre-planned condition [66, 80]. More importantly, individuals experience greater peak knee valgus moments during unplanned conditions than during pre-planned conditions [21, 66]. Although the magnitude of ACL strain increases, it is currently not clear whether the movement execution varies enough to be considered a different movement strategy defined by the distribution of kinetics (i.e., shifting from “hip” to “knee-dominant”) and this requires further investigation.

**Fig. 5:**
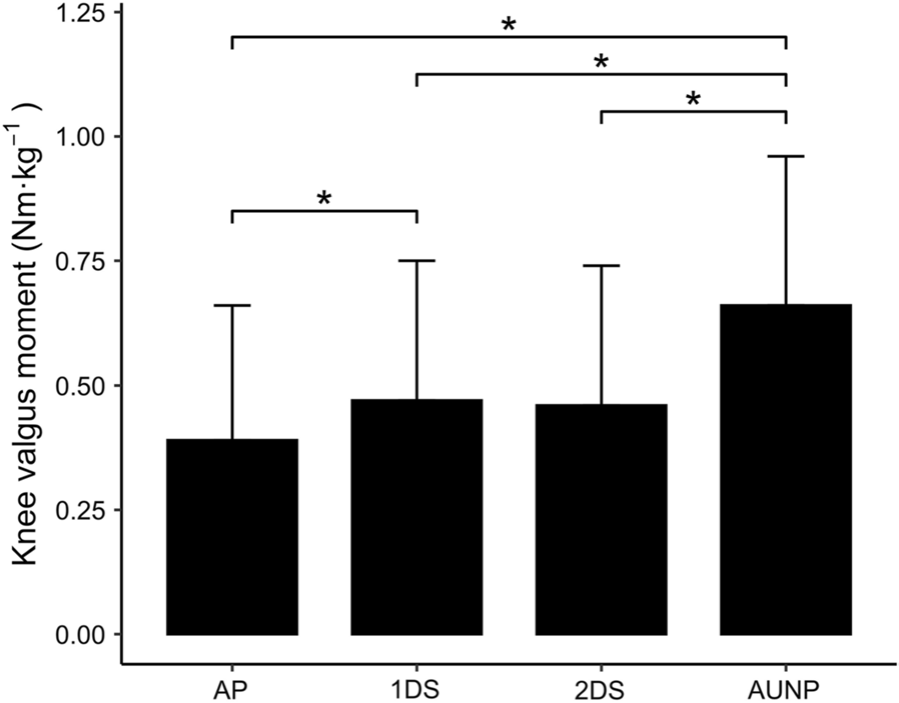
Knee valgus moments in response to 4 different sidestep conditions. AP = Arrow planned (pre-planned); 1DS = One-defender scenario (specific information); 2DS = Two-defender scenario (specific information); AUNP = Arrow unplanned (generic information). * Significantly different at *p* ≤ 0.05. Data from Lee et al. [18]

**Fig. 6:**
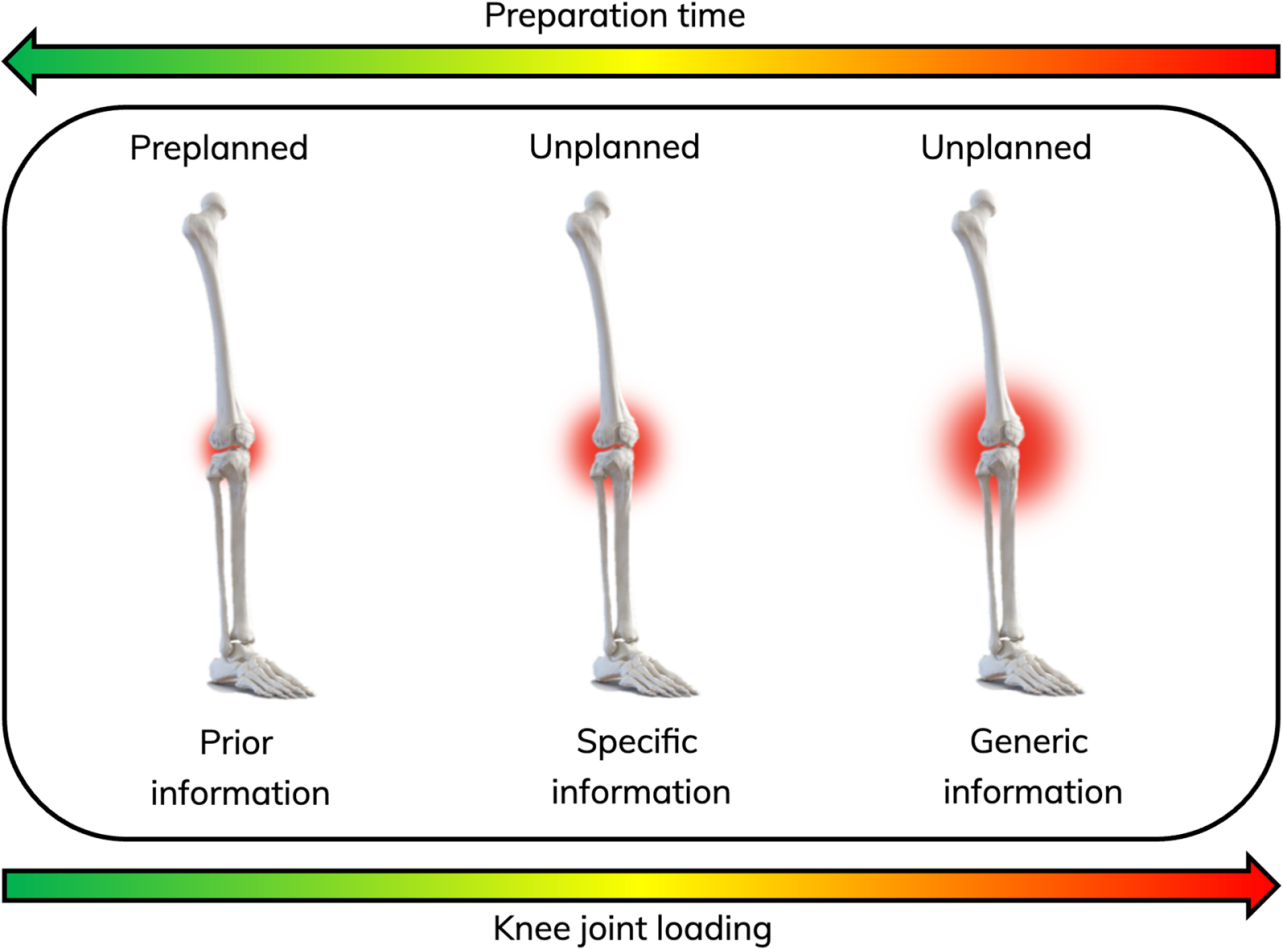
Knee joint loading continuum in response to different information sources. Data from Lee et al. [18]

Since different stimuli or information reduce or increase the time available to prepare for sidestepping, the result is a change in the demands on the knee joint within the same broadly categorized movement strategy of sidestepping (Fig. 5). Therefore, a spatiotemporal continuum is one way to consider the relationship between time available and knee joint loading (e.g., knee valgus moment). Pre-planned sidestepping can be regarded as at one end of the continuum with minimal spatiotemporal restriction and contrasts with unplanned sidestepping in response to generic information with no anticipatory cues to draw from to increase the opportunity for earlier anticipation. When specific information, such as a 3D projection of one or more opponents, is exploited, the opportunity to anticipate increases, providing more time to prepare for the upcoming sidestepping [21]. In such situations, when the opponents are not attempting to deceive, athletes tend to have relatively good anticipation accuracies with few response corrections [81], which translates to reduced ACL strain compared to movement adaptations following generic information (Fig. 6). Despite such biomechanical insights, researchers have suggested periodizing training from unplanned scenarios to generic information and specific information [82]. This suggests the previous order of progression could be questioned when a progressive exposure joint loading is necessary to minimize injury risk or return an athlete to performance following injury and is contrary to the loading continuum.

### What are Additional Considerations for Using Anticipatory Information as a Constraint for a Loading Continuum?

Although the opportunity to anticipate from the information provided decreases the demands on the knee joint at a group level, this does not guarantee that injury risk reduction occurs at an individual level as the skill of anticipation differs between athletes. This heterogeneity in the skill of anticipation, which is associated with decision-making processes [83, 84], is not only apparent between different amounts of sport-specific expertise [85, 86] but also present within groups of elite athletes [87]. In general, athletes with poorer anticipation skills have less time to prepare for sidestepping, placing them more likely in “worst-case” scenarios with concomitant high-impact demands on the knee joint [21]. However, while “early” anticipation and decision-making might be considered beneficial, earlier response actions have been found to increase the likelihood of being deceived [81, 88] while giving the opponent to the opportunity to change their movement [89]. Therefore, it appears to be particularly beneficial to execute movements at the last moment possible, so one can acquire as much information as possible while tolerating the demands imposed or force expressed to execute a successful performance. Moving at the right time is much more important than moving as early as possible. Skilled athletes initiate their movements later, which increases the outcome accuracy and success as there is less susceptibility to deception and superior motor capacities enable them to tolerate the imposing demands [81, 90]. Further, evidence suggests that skilled athletes seem to make better choices rather later, instead of earlier, when tested with representative task designs [88, 91, 92], indicating that the quality of a decision might be of higher relevance than the required time to decide [93]. This is in accordance with the speed–accuracy trade-off paradigm, suggesting that a more time-consuming evaluation of a situation can lead to higher success rates and fewer errors [88, 91, 92, 94, 95]. However, even athletes with well-trained anticipation and decision-making skills may experience in situ scenarios where they cannot rely on those skills (i.e., due to obscured vision, an opponent’s use of deception and disguise, or stochastic ball bounce) and therefore experience “worst-case” scenarios. Thus, exposing athletes to unplanned sidestepping situations that include generic information may further increase their load tolerance or provide specific development of motor capacity. In contrast, practice conditions with an opponent not using deception or disguise might not result in tissue strain sufficient to elicit adaptation, especially in athletes already skilled in anticipation and decision-making [85, 86]. In such scenarios, reacting to generic information (i.e., flashing lights or verbal cues) may provide an appropriate overload to further increase motor capacities.

## Perceptual–Cognitive Skill and Improving Perceptual–Motor Skill

### Will Improvements in Perceptual–Cognitive Skill Facilitate Perceptual–Motor Performance?

Understanding the isolated and interacting intrinsic and extrinsic constraints is crucial when attempting to alter an athlete’s movement, enhance athletic performance, and reduce injury risk, especially during “worst-case” scenarios. As sidestepping is ultimately a perceptual–motor skill, research efforts have aimed to improve perceptual–cognitive skills and motor capacities with targeted interventions. Specific to the former, an extensive body of literature has demonstrated that elite athletes perform better than sub-elite athletes on tests of perceptual–cognitive skills [96]. It follows that improving the interrelated perceptual–cognitive skills such as gaze control, anticipation, and decision-making may enable athletes to better adapt to the demanding spatiotemporal constraints that are replete in elite sports, often with the goal of providing athletes with more preparation time prior to “worst-case” scenarios. However, the efficacy of training perceptual–cognitive skills in isolation in non-representative settings to improve in situ performance remains questionable [78, 97, 98].

The continuum of sidestepping situations described in the previous section also reflects a continuum of training practices, which can be further considered in the context of extant perceptual–cognitive training frameworks [79, 99]. To this end, in the perceptual–cognitive skill literature, there has been an increased tendency for researchers to emphasize that if a training environment does not adequately sample characteristics from the sport performance environment, this may have a limiting impact on the efficacy of perceptual–cognitive skill interventions. For instance, in the representative learning design framework of Pinder et al. [100], these authors emphasized two considerations when evaluating the suitability of training conditions, namely functionality and action fidelity. Functionality emphasizes that performers should regulate their actions in learning contexts relative to information in the performance environment. By way of example, researchers have questioned the suitability of ball-projection machines to create training conditions for athletes in fast-ball interception sports such as cricket [100] and tennis [101]. Further, action fidelity considers whether a performer’s responses (e.g., actions or decisions) are equivalent between the training environment and the performance setting. For instance, Maloney et al. [102] demonstrated that motor actions in the sport of taekwondo differ between training and competition, as the former failed to adequately create the pressure, arousal, and mental challenge associated with competition [103]. This highlights that training any perceptual–motor skills without adequately facilitating an appropriate level of functionality and action fidelity that resemble those experienced in situ inhibits how an individual perceives and acts and thus fundamentally limits a transfer to in situ scenarios.

Video training is a particular mode of perceptual–cognitive training that is critiqued for lacking a representative learning design [100]. This mode of practice has commonly been studied in the literature, partly because video training may enhance an athlete’s perceptual–cognitive skill without placing any additional physical stressor on an athlete beyond regular training and competition [16]. However, the potential benefits of video training have been questioned [97] as evidence points to the fact that video and field-based measures of anticipation and decision-making appear to capture different elements of these respective skills [104]. For example, in a study of skilled football players, van Maarseveen et al. [105] reported that on-field anticipation and decision-making performances were not predicted by video-based tests designed to measure the equivalent perceptual–cognitive skills. These findings lend support to the perspective of van der Kamp et al. [106]. They integrated the ecological approach with the visual perception of Gibson [76] alongside the neuro-anatomical perspective of Milner and Goodale [107, 108]. In short, van der Kamp et al. [108] and colleagues emphasized that to study the complementary perception–action (i.e., ventral and dorsal systems [108]:) process that is critical to perceptual–cognitive skill, it is essential for researchers to study the control of movements in real time relative to game context (e.g., the actions of an opponent and ball-flight) instead of asking participants to make simulated responses to a video. The implication for training is that while the video may enable the systematic control of information, such training design does not allow athletes to interact with and influence the ever-changing environment of real-time sports situations [97]. Thus, although some evidence indicates that high-skilled athletes may benefit from video-only training [109, 110], questions about the efficacy of this training mode for improving perceptual–cognitive skills that are transferred to in situ scenarios remain largely unanswered [97].

Small-sided games (SSG) reflect another popular training method that has the potential to not only improve perceptual–cognitive skill—due to the perception–action processes involved—in tandem with physical and technical qualities. Variations of SSG, such as modifying pitch dimensions and player numbers, can increase spatiotemporal demands and the frequency of opportunities for anticipation and decision-making. Indeed, the reactive agility test (RAT) performance, which aims to quantify the decision-making time and change of direction speed, has been shown to increase after interventions with SSG [111, 112, 113]. However, while these results appear to be beneficial at first glance, the RAT is reliant on video-based tests. Responses to video-based tests differ from on-field decision-making [105] and elicit a different action than an equivalent on-field condition of the same situation [104]. Therefore, it remains speculative whether improvements in RAT are a valid and reliable surrogate of in situ anticipation and decision-making. Since perceptual–cognitive skills are suggested to be task and environment-specific [114, 115], and variations of SSG setup affect divergence in player actions and positioning [116], practitioners need to be mindful when designing SSG to improve specific in situ perceptual–cognitive skills. Future research is required to elaborate on how different methods like video training and SSG protocols develop in situ perceptual–cognitive skills and support anticipation performance in “worst-case” scenarios. For example, it has been proposed that practitioners may benefit from adapting strategies nested in the ecological dynamics and constraints-led frameworks to help facilitate technical-tactical development [117]. As such, conceptualizing drills within sports-specific practice that promote movement adaptability (degeneracy) within representative learning environments may offer more potential to develop perceptual–cognitive skills that transfer to in situ scenarios. While research is needed to support such suggestions, designing technical-tactical training in the above way could mean that the allocated time for strength and conditioning practice should prioritize physical preparedness.

## Conclusion and Practical Applications

Individual constraints, such as motor capacity (i.e., strength), dexterity, or available range of motion, dictate the available solution space and affect the emergent movement strategy when sidestepping. With increasing task demands, fewer movement strategies are available to athletes, potentially catalyzing a repeated utilization of the same dominant movement strategies. Therefore, having the motor capacity to adapt and utilize different movement strategies increases the likelihood of success “despite” the scenario or position that the athlete is in. When increasing motor capacities, practitioners need to concomitantly facilitate the (re)calibration to the newfound strength through explorative practice to adequately adopt new movement solutions for a motor task. In the current article, we have proposed that the ability to adapt is especially pertinent during “worst-case” scenarios, where the imposed demands can surpass an individual’s load tolerance and increase injury risk. Training for such “worst-case” scenarios can be facilitated when the time available to anticipate and prepare for sidestepping is insufficient, such as when acting relative to generic information, including the sudden onset of a flashing light or verbal cues. Despite attempts to improve perceptual–cognitive skills, which enable better anticipation and decision-making, conclusive evidence is still lacking on whether a transfer to in situ scenarios is fully supported. Consequently, practitioners should strive to increase an athlete’s load tolerance and broaden the possible solution space to minimize injury risk, particularly in “worst-case” scenarios. Traditional resistance and plyometric training methods can thereby increase single- and multi-joint strength and facilitate load tolerance and injury resiliency. However, further research is needed to evaluate the efficacy of deliberately imposing task constraints to alter an athlete's habitual execution strategy execution variability.

Understanding how constraints influence an athlete’s movement strategies and the associated demands on the joint(s) can help practitioners design appropriate practice conditions to prepare athletes for “worst-case” scenarios (see Additional file 1: video). Changing the practice conditions will require the athlete to explore different movement strategies and change the magnitude and distribution of joint loading. Knee joint loading (e.g., knee valgus moment) increases with decreasing preparation time. While preparation time is dictated by an athlete’s anticipation and decision-making qualities, future work is still needed to fully understand how to improve off or on-field-based perceptual–cognitive skills that transfer to in situ scenarios. Developing single- and multi-joint strength facilitates a broad base of execution strategies and ensures an increased tolerance threshold against imposing loads. Since different tissues adapt at different rates, we recommend practitioners progressively introduce more demanding drills and incorporate appropriate deload phases to allow for musculoskeletal adaptations to manifest. Creating variable practice conditions encourages exploration and enables athletes to (re)calibrate and learn how to use their improved motor capacity.

When implementing perception–action practice conditions, practitioners will be affecting an athlete’s preparation time and therefore changing the imposed demand. This becomes the ideological shift for practitioners on the purpose of perception–action practice. There is more evidence to understand that perception–action training affects the demands on the joint(s) than it being a method of perceptual–cognitive skill training that transfers to in situ performance. Therefore, practice conditions should be implemented using a task-constraint continuum with the source of information dictating the amount of time available to the athlete and consequently changing the imposed loading. A reduction to the “worst-case” scenario or no preparatory timing elicits high demands on the knee joint, building an athlete’s physical load tolerance if done systematically and progressively.

## Supplementary Information

**Additional file 1**: Exemplary sidestep progression with increasing demands on the knee joint with and without the addition of spatiotemporal constraints.

## Data Availability

Not applicable.
